# The Distribution of PSA Age-Specific Profiles in Healthy Irish Men between 20 and 70

**DOI:** 10.5402/2012/832109

**Published:** 2012-07-26

**Authors:** R. G. Casey, P. K. Hegarty, R. Conroy, D. Rea, M. R. Butler, R. Grainger, Ted McDermott, J. A Thornhill

**Affiliations:** ^1^The Adelaide and Meath Hospital-Dublin, Incorporating The National Children's Hospital, Tallaght, Dublin 24, Ireland; ^2^Department of Biostatistics, Royal College of Surgeons in Ireland, 123 Street Stephen's Green, Dublin 2, Ireland; ^3^Marjorie White Flynn, Department of Urology, The Adelaide and Meath Hospital-Dublin, Incorporating The National Children's Hospital, Tallaght, Dublin 24, Ireland

## Abstract

*Background*. Ireland is estimated to have the highest European incidence rate of prostate cancer (Pca) in 2006 which will increase by 275% by 2025. This study aimed to determine PSA cutoff values in different age groups of healthy male patients without Pca. *Methods*. 660 men in a pilot men's health programme, aged 18–67, had PSA assayed. Men were grouped into 8 age groups at 5-year intervals: 30–34, 35–39, 40–44, 45–49, 50–54, 55–59, 60–64, and 64–70. *Results*. Linear regression demonstrates a PSA velocity of 0.024 ng/ml/year. The 95% confidence interval demonstrates a near flat line of PSA values from age 20 to 50 and rises after. When transformed logarithmically, PSA correlates highly with expected values from the normal distribution (0.98). A fractional polynomial quantile regression model was used to predict median and 95th percentile for PSA as follows: 30–34 (0.73, 1.57), 35–39 (0.71, 1.65), 40–44 (0.73, 1.85), 45–49 (0.78, 2.17), 50–54 (0.88, 2.63), 55–59 (1.01, 3.25), 60–64 (1.20, 4.02), and 64–70 (1.43, 4.96). *Conclusions*. PSA levels are similar to other racial groups but not as high as US Caucasians until 65 years. These data define the predicted PSA for the Irish population and provide a reference for future screening programmes.

## 1. Introduction

 Prostate cancer (PCa) is the most commonly diagnosed life-threatening cancer amongst men in many industrialised nations. Ireland was estimated to have had the highest PCa incidence rate in Europe in 2006 and incidence is expected to have increased by 275% between 2000 and 2025, given current trends and our ageing population. In contrast, the rate of PCa mortality among Irish men was estimated to be the tenth highest in Europe [1, 2].

Prostate-specific antigen (PSA) is a serine esterase, which is produced almost exclusively by the prostatic epithelium [3]. Its use as a tumour marker has been established for over twenty years [4]. However, it is not specific for PCa and has a high false positive rate when used as a screening tool [5].

The normal upper limit of PSA, 4.0, is not always accurate for all ages [6]. Papers have suggested that age-specific cutoff values for PSA screening are better compared than the currently used single cut-off of 4.0 ng/mL [7]. PSA may increase with prostatic hyperplasia; therefore, one would expect that the PSA level should be lower in younger men. The currently used single cut-off of 4.0 ng/mL underestimates the cancer risk in younger patients and may also result in unnecessary biopsies in older men with benign prostatic hyperplasia [8, 9]. However controversy surrounds this definition of normal PSA level, most recently by the Prostate Cancer Prevention Trial (PCPT) which demonstrated a 17% of men with PSA <2.0 ng/mL were found to be harbouring PCa (11.8% were high grade) [9].

Age-specific reference ranges for PSA were first presented from a community-based population of 471 healthy American white men by Oesterling [8]. There is increasing concern over the general applicability of those reference ranges. Different races have their own reference ranges because of the influence of geographic and ethnic differences [10]. Similar studies have presented a number of different groups of men [10–15], but no large-scale study has been conducted in Irish men.

Ireland is a country with a largely homogenous population of Celtic extraction with a traditionally low rate of immigration and would benefit from assessment of its particular PSA profile. Up to now, we have been using internationally established ranges of PSA based on Oesterlings study [8].

To optimize the application of the PSA test in Ireland, this study was performed to determine cutoff values in different age groups based on clinical evaluation of a large number of healthy male patients undergoing health examinations without a diagnosis of PCa.

## 2. Materials and Methods

As part of a pilot urological health promotion programme, all male employees in a multibranch bank were invited to undergo screening. This was advertised to the bank's 5000 employees using the intranet system. Within 24 hours, the recruitment was closed at 660 subjects, between 18 and 67 years of age. All patients had no clinical evidence of PCa. This was assessed by questionnaire and digital rectal examination.

Men were grouped into 8 age groups at 5-year intervals: 30–34, 35–39, 40–44, 45–49, 50–54, 55–59, 60–64, and 64–70. Blood samples were collected from each patient and total PSA was measured using a tumour marker assay analyser, using the IMx assay (Abbot Laboratories, Chicago).

All data was anonymised but linked with a patient identifier and stored securely in the Urology department on a password-protected computer.

## 3. Statistical Analysis

 Data were analysed using Stata Release 7. Because the focus of the analysis was on determining reference values for the population, quantile regression was used, as implemented in Stata's qreg procedure. Unlike conventional least-squares regression, which predicts the mean value of the variable of interest, quantile regression can be used to predict a specific percentile. In this case, we chose the median and the 95th percentile as providing the most useful information about the distribution of PSA by age in the Irish population. Fractional polynomial regression models were used to allow the relationship between age and PSA to depart from linearity, using an algorythm originally developed by Royston [5] and subsequently implemented in Stata's fracpoly procedure. Fractional polynomials are ideally suited to problems where the objective is correctly modelling the shape of a relationship, making them a valuable method in the calculation of population norms.

## 4. Results

The mean age of the patients was 58 (range 25–70). In the entire cohort, the mean PSA level was 1.7 (median 0.9 range 3–83).

**Figure 1:**
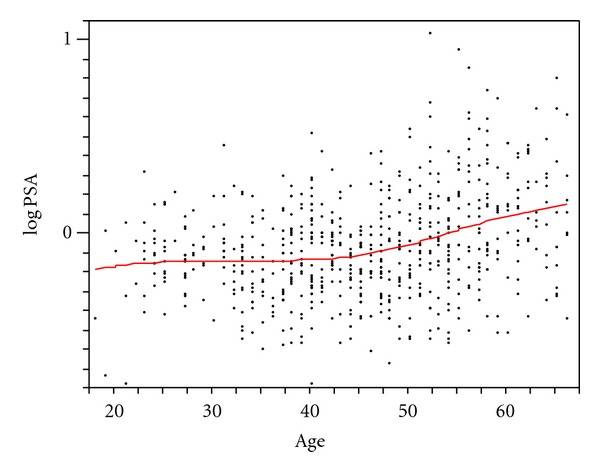
The graph shows the relationship between age and PSA (on a log scale) with a cubic spline with lambda set to 10,000.

The distribution of PSA was approximately lognormal (Figure 1). However, even when log-transformed, the relationship between PSA and age departed slightly from strict linearity. A LOESS smoother curve that fitted 99% of the observations showed that the relationship of PSA and age became steeper as age advanced. Fractional polynomial quantile regression was used to estimate the median and 95th percentiles of the PSA distribution as a function of age, together with their associated 95% confidience intervals. These estimates are shown in Table 1 at five-year-age intervals from 25 to 65.

As can be seen from Table 1, median PSA begins to rise between age 40 and 50 and reaches a predicted 1 ng/mL at age 55. The 95th centile in a 60-year-old man is 4 ng/mL, but rises to just under 5 ng/mL at age 65.

## 5. Discussion

Age-specific reference ranges were introduced to increase the specificity and sensitivity of a PSA result. Cross-sectional data as derived from the study of men in Olmsted County Minnesota suggests that serum PSA rises with advancing age [6]. In the Baltimore longitudinal study of aging, the observed median increase in serum PSA of men in the absence of prostatic disease was 0.03 ng/mL/yr. Consequently, the age-specific reference ranges must undergo commensurate adjustments. The recommended age-specific reference ranges Oesterling proposed based on the Olmsted County data for serum PSA are 0–2.5 ng/mL for men aged 40–49 yrs, 0–3.5 ng/mL for men aged 50–59 yrs, 0–4.5 ng/mL for men aged 60–69 yrs, and 0–6.5 ng/mL for men aged 70–79 yrs [7]. Our results are broadly in agreement with these ranges, though the lack of older participants made it impossible for us to calculate predicted values beyond age 65. The original hypothesis proposed by Oesterling was that age-specific reference ranges for serum PSA would potentially detect earlier organ-confined prostate cancer in younger men at a time when the tumours are potentially more susceptible to cure (increased sensitivity), while at the same time detect less cancers in older men who might have clinically insignificant tumours or have a less than 10-year life expectancy (increased specificity).

The use of younger subjects in this study demonstrates PSA levels in a group in which PCa and benign prostatic hyperplasia are less prevalent. The PSA levels change very little between the ages of twenty and fifty. It is interesting that the median PSA does not rise until this age given that there is histological evidence of BPH in men in their thirties [8]. The low prevalence of prostatic pathology in men in their thirties may account for the rising predicted PSA at the 95th centile from age 35, whereas the median value remains unchanged for a further ten years. The observed increases in PSA may reflect the increasing prevalence of pathology rather than a physiological process of ageing.

Ideally, the use of age-specific reference ranges should use reference table's specific to the normal PSA profile of the population. It has been found that if age-specific reference ranges derived from white populations were applied to blacks, over 40% of cases of PCa would be missed [16]. Similarly, in Jordanian patients they exhibit higher total PSA values and those are more widely distributed than those of previous reports, particularly in older men [15].

Potential bias exists in this study as the men who volunteered for inclusion firstly are from a banking background which may represent a higher urban socioeconomic class and therefore knowledge base as demonstrated previously (Casey RG, Prostate cancer knowledge in Irish men, unpublished work). Secondly, there may also be a “volunteer bias” as men who volunteered may be more health conscious.

Regression analysis suggests that a concomitant increase in prostatic volume may account for some of the age-related increases in serum PSA. Other suggested factors include (a) subclinical/clinical prostatitis, (b) intermittent bouts of prostatic ischaemia/infarction [17], and (c) the presence of PCa that cannot be detected by currently available methods. Furthermore, as men age their prostates may become more permeable due to the breakdown of the physiological barriers, which normally prevent PSA entering the general circulation [18]. We suggest that the stable PSA levels between ages of 20 and 50 reflect the relative absence of disease, and that increases thereafter are due to the development of PCa or BPH. Our reference ranges are purely statistical and cannot strictly define men's disease status without prostate histology or radical prostatectomy specimens. Another approach would be to repeat the PSA in all 660 men at intervals to define PSA velocities for each individual and observe the cohort for eventual diagnosis of PCa.

We confirmed the relation between age and PSA level but the present median PSA levels were lower (Table 1) than those reported by Oesterling and in accordance with the reports by Thompson et al. [9] and Loeb et al. [6]. Notably, this relation between PSA level and age is less pronounced in this contemporary cohort than it was in the men studied by Oesterling [8] 12 years ago.

This phenomenon can be explained by PSA-based early detection efforts, which eliminated those men with high PSA values. The present findings are unique in the origin of the population. They indicate that PSA levels require reassessment in countries other than the USA, where screening has been enforced with strength and enthusiasm.

The present study demonstrates PSA levels similar to other racial groups, meaning that international standards are applicable to this population. However, from Table 2 it can be noted that PSA levels in Irish men are not as high as Western US Caucasians until the age of 65 years and lie somewhere midway between Western and Asian level prior to this. Furthermore, it provides a reference of predicted PSA levels for Irish males up to the age of seventy. Our results may help us to increase the sensitivity and specificity in detection of PCa in different age groups. Thus, PSA levels should be reassessed in other countries/continents where the detection of PCa and its genetic make-up may differ from the USA.

## Figures and Tables

**Table 1:** Predicted median and 95th centile of PSA, 95% confidence intervals, for age groups of 25 to 69.

Age (years)	Predicted median	95% CI	Predicted 95%ile	95% CI
25–29	0.77	0.65–0.89	1.59	0.58–2.60
30–34	0.73	0.65–0.81	1.57	0.86–2.29
35–39	0.71	0.65–0.78	1.65	1.11–2.20
40–44	0.73	0.67–0.79	1.85	1.35–2.34
45–49	0.78	0.72–0.84	2.17	1.67–2.67
50–54	0.88	0.82–0.94	2.63	2.14–3.13
55–59	1.01	0.95–1.07	3.25	2.72–3.77
60–64	1.20	1.11–1.28	4.02	3.31–4.73
65–69	1.43	1.28–1.57	4.96	3.83–6.08

CI: Confidence interval.

**Table 2:** Comparative predicted 95%iles for different groups of men.

Age	White US [19]	African US [20]	Asian US [20]	Taiwanese [21]	Japanese [10]	Korean [12]	Jordanian [15]	Irish
25–29	—	—	—	1.86	—	—	**1.59**
30–34	—	—	—	1.85	—	1.8	2.3
35–39	—	—	—	1.85	—	1.8	2.9
40–44	2.5	2.7	2.0	2.17	2.0	2.0	3.15
45–49	2.5	2.7	2.0	2.17	2.0	2.0	3.75
50–54	3.5	4.4	3.5	3.32	3.0	2.5	3.8
55–59	3.5	4.4	3.5	3.32	3.0	2.5	3.76
60–64	4.5	6.7	4.5	5.01	4.0	3.9	4.3
65–69	4.5	6.7	4.5	5.01	4.0	3.9	—
70–79	6.5	7.7	6.5	6.2	5.0	6.3	—
